# Wearing an Activating Spinal Orthosis and Physical Training in Women With Osteoporosis and Back Pain: A Postintervention Follow-Up Study

**DOI:** 10.1016/j.arrct.2021.100154

**Published:** 2021-08-27

**Authors:** Christina Kaijser Alin, Ann-Charlotte Grahn-Kronhed, Elin Uzunel, Helena Salminen

**Affiliations:** a Division of Family Medicine and Primary Care, Department of Neurobiology, Care Sciences, and Society, Karolinska Institutet, Solna, Sweden; b Rehab Väst, Local Health Care Services in the West of Östergötland, Mjölby, Sweden; c Division of Prevention, Rehabilitation and Community Medicine, Department of Health, Medicine and Caring Sciences, Linköping University, Linköping, Sweden; d Academic Primary Healthcare Centre Stockholm, Stockholm, Sweden

**Keywords:** Exercise, Kyphosis, Osteoporosis, Rehabilitation, Spinal orthosis, RCT, randomized controlled trial

## Abstract

**Objective:**

To assess the duration of benefits on back pain and back extensor strength in women with osteoporosis who had previously participated in a randomized controlled trial (RCT) involving either exercise or wearing a spinal orthosis.

**Design:**

A 6-month postintervention follow-up of women who were involved in the interventions in the RCT.

**Setting:**

The study was conducted in a primary health care center in Stockholm, Sweden.

**Participants:**

In this follow-up study 31 women participated in the spinal orthosis group, and 31 women participated in the exercise group, with a median age of 76 years in both groups (N=62). All women were diagnosed as having osteoporosis, had back pain with or without vertebral fracture, and were 60 years or older, which were the inclusion criteria in the RCT.

**Interventions:**

The participants received no controlled supervision. The spinal orthosis group was asked to wear the orthosis, and the training group was asked to follow an exercise program for another 6 months voluntarily.

**Main Outcome Measures:**

Back extensor strength was measured with a computerized device; back pain was estimated by the visual analog scale and by Borg CR-10.

**Results:**

After 6 months there were no significant differences between the groups in back extensor strength or back pain. Analyses within the groups showed that achieved results during 6 months intervention in the RCT were maintained after 6 months of voluntary use of the spinal orthosis and training. In the spinal orthosis group, back extensor strength mean was 81.7 N, and back pain median was 3 mm. In the training group back extensor strength mean was 72.8 N, and back pain median was 3 mm. There were no changes for any other measurements performed.

**Conclusions:**

Voluntary use of the spinal orthosis or exercise during a 6-month follow-up period maintained the increase in back extensor muscle strength obtained during the RCT. Estimation of back pain was not influenced. This indicates that the women had continued to use the spinal orthosis and exercise.

Older women with osteoporosis and back pain are a common group of patients in primary health care. Training of back extensor muscles is an important part of the treatment in these patients. An increase in back extensor strength has in previous studies shown to decrease problems with back pain and also to make daily activities easier.^1^, ^2^, ^3^ It has also been shown that vertebral fractures can be prevented by activating and training back extensor muscles.^4^, ^5^, ^6^

Training of back extensor muscles can be performed either by individually tailored training in a gym or by training at home according to an individually tailored exercise program. These 2 different training methods have shown a positive effect on back muscle extensor strength.^7^

An activating spinal orthosis has been developed that allows some mobility of the back.^6^ A steel rail is adapted to the back and is plugged into a compartment of the orthosis. There are straps around the shoulders, and the lower part of the orthosis is fastened over the pelvis.

When used, it provides a continuous activation of the back extensor muscles. The activating spinal orthosis has been used in rehabilitation of patients who have osteoporosis, vertebral fractures, and back pain. Several studies have shown that treatment with the activating spinal orthosis increases muscle strength in the back extensors along with decreasing back pain.^3^^,^^8^^,^^9^, ^10^, ^11^, ^12^, ^13^ It has also been shown that regular follow-up over a longer period of time may be needed regarding walking aids, considering that there may have been changes in the individual's physical condition and/or because of the environmental factors.^14^^,^^15^

The aim of this study was to explore how obtained results would be influenced by voluntary use of an activating spinal orthosis or training during a postintervention period of 6 months.

## Methods

### Design

**Fig 1:**
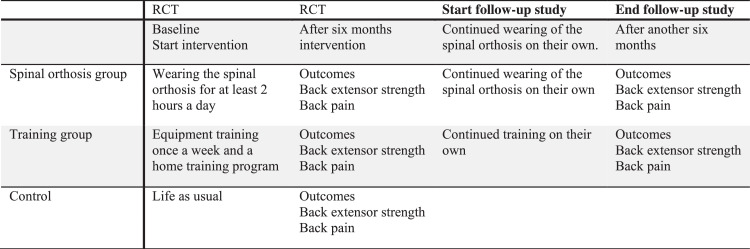
Study design of the present postintervention follow-up study and the RCT.

This study was an extended postintervention follow-up study of a randomized controlled trial (RCT) where 113 women were randomized to 3 arms: an activating spinal orthosis group (n=38) who wore an activating spinal orthosis for at least 2 h/d, an equipment training group (n=38) who exercised once a week, and a control group (n=37). The women who participated in the study received comprehensive information and gave their written informed consent. The intervention period lasted for 6 months.^12^ The 2 intervention arms were followed for another 6 months in this follow-up study (fig 1).

### Participants and context

**Fig 2:**
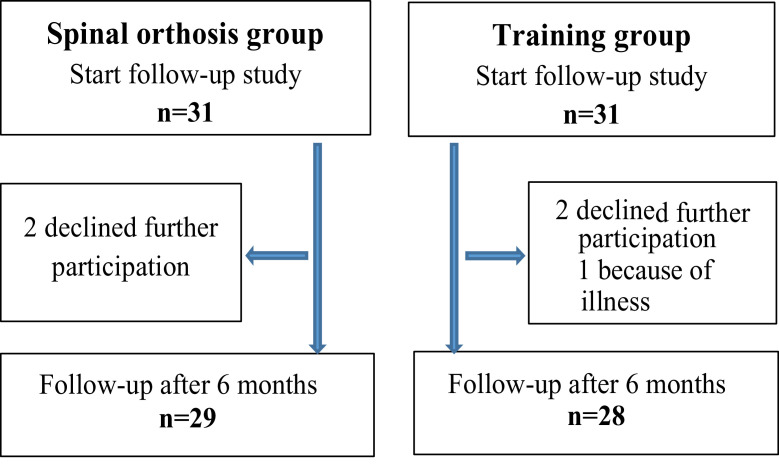
Flowchart of the participants in the postintervention follow-up study.

Inclusion criteria in this follow-up study were women who had previously participated and completed a 6-month intervention period in the RCT: 31 women in the activating spinal orthosis group and 31 women in the equipment training group^12^ (fig 2). Inclusion criteria to the RCT were women, diagnosed osteoporosis, back pain with or without vertebral fractures, and age of 60 years or older. This postintervention follow-up study was conducted from November 2012 to June 2015, in 4 rounds. The first follow-up started in May 2013, the second in February 2014, the third in October 2014, and the last one in June 2015. The participants were examined by a physiotherapist or a physician at a rehabilitation center in primary health care at Sabbatsberg Hospital in Stockholm City.

In the present follow-up study, the participating women were asked to return after 6 months for a follow-up visit, where they were retested and were asked about the training and use of the spinal orthosis. During the 6 months postintervention period no controlled supervision took place. However, the women were asked to use the activating spinal orthosis Spinomed^a^ based on their needs. They could individualize the time and the frequency wearing it, but they did not report time wearing the orthosis. Previously in the RCT, the women were asked to wear the orthosis for a total of 2 hours or more per day, and these 2 hours could be divided into shorter periods during the day.^12^ The participating women had no planned follow-ups with orthopedic technicians. However, if there was any problem with the orthosis, contacts could be made. The women in the training group exercised completely on their own, and they did not report in an exercise diary.

In the present follow-up study, the participating women who had joined the training group continued with their home training program but had no organized training activities. Previously in the RCT, the participants had followed an equipment training program led by a physiotherapist once a week and a home exercise program at least 4 times a week. The exercises focused on increasing muscle strength of the back and of the legs along with improving posture and balance. In the home exercise program, the back extensor strength was trained with rubber bands 2 × 25 times, with dumbbells 2 × 10 times, and by back raising (lying on the abdomen) 1 × 10 times. Balance was trained by tandem standing, 1-leg standing 30 seconds with eyes open and closed and 1-leg standing while brushing one's teeth. The muscle strength of the legs was trained with quick chair stand raising 10-15 times.^12^ The participants in the control group were excluded because the Ethical Review Board considered it not justifiable to have patients with diagnosed osteoporosis without any active intervention for a further 6 months.

### Primary outcomes

The primary outcomes of the present study were back pain and back extensor strength. Back pain was measured by the visual analog scale (VAS), where no pain was rated as 0 mm, and worst possible pain as was rated as 100 mm and by Borg CR-10 (0-10).^16^, ^17^, ^18^ An overall assessment of back pain for the previous week was scored as well as present back pain. Isometric back extensor strength was determined with the computerized device DigiMax.^b^ Participants were asked to press the upper part of the body against a plate for 6 seconds sitting in a fixed standardized position, with 90 degrees in hip and knee fixed by a seatbelt around the chest and hip. The force that was developed was presented in Newtons, both as a mean and as a maximum value at any time during the 6 seconds.

### Secondary outcomes

Secondary outcomes were spinal curvature and balance. The spinal curvature was measured by the Flexicurve ruler,^c^ which is molded to the curve of the spine with the participant in an upright position.^19^, ^20^, ^21^ The kyphotic index is calculated as “the kyphosis width divided by the length times 100.”^12^^(p4)^ A clinically relevant cutoff point for the presence of hyperkyphosis was set to a kyphotic index ≥13.^22^

Balance performance was assessed by standing on 1 leg, tandem standing, and Romberg's test, with eyes open and closed. The results were presented in seconds with a maximum of 30 seconds. Tandem gait forward and tandem gait backward were measured on a line where the number of steps was counted with a maximum of 15 steps. Timed gait speed for 30 m was measured.

### Other measurements

Present body height was measured in centimeters by a stadiometer in a standing position with the woman's heels against the wall. Weight was measured in kilograms. Hand grip strength was measured by the Jamar^d^ dynamometer in kilograms both of the dominant and the nondominant hand.^23^ Forced vital capacity was assessed by spirometry.^e^ Data were collected concerning history of fractures. Questions of the use of medication concerning bone-specific drugs and calcium in combination with vitamin D were answered. Self-rated current perceived health was estimated using the EuroQol visual analog scale (0-100mm) with the endpoints “best imaginable state” (100) and “worst imaginable state” (0).^24^^,^^25^ Smoking and time spent outdoors at least 30 min/d, physical activity, or a walk at least 3-5 d/wk were collected. To investigate the presence of vertebral fractures a sagittal radiography was taken of the thoracic and lumbar spine at baseline in the RCT.

### Statistical analysis

A paired *t* test was used to analyze changes in the groups between the start of the postintervention follow-up study and after 6 months and were reported as means and SDs for normally distributed continuous variables. Wilcoxon signed-rank test was used for skewed distribution, and results were reported as medians with interquartile range. We analyzed whether there was a difference between the groups at the postintervention follow-up start and after 6 months. For normally distributed continuous variables Student *t* test was used, and results were reported as means and SDs. For variables with a skewed distribution, Mann-Whitney *U* test was used, and results were reported as medians with interquartile range. Significance levels below 5% were considered significant. The data were analyzed using the Stata version 14.^f^

### Ethical considerations

Ethical approval was obtained from the Regional Ethical Review Board of Stockholm (Dnr 2011/142-31/3).

## Results

A total of 57 women completed the follow-up study. At study start, there were 31 women in each group. At the end of the study there were 29 women in the spinal orthosis group, and 2 women declined further participation. In the training group there were 28 women, 2 women declined further participation, and 1 woman could not participate because of illness. Median age was 76 years (range, 66-84y) in both groups. The particular characteristics of the study participants at the start of the follow-up study are shown in table 1. Almost half of the women in each group had a kyphotic index ≥13, and about 50% of the women were treated with bone-specific drugs. The percentage of women treated with calcium and vitamin D was around 90% in both groups. More than three-quarters of the women spent >30 min/d outdoors. Two-thirds of the women in the training group and half of the women in the spinal orthosis group were physically active or went for a walk at least 3-5 d/wk. Of all participating women in the study, only 1 woman in the training group smoked. In the spinal orthosis group there was no previous hip fracture, whereas 2 women in the training group reported a previous hip fracture when they entered the study. In each group, one-quarter of the women reported a previous forearm fracture. Radiographs of the back showed that almost half of the women in each group had sustained 1 or several vertebral fractures. At the start of the postintervention follow-up study there were significant differences between the groups on 3 variables: weight, 1-leg standing with eyes open, and tandem standing with eyes closed, where the participants in the training group showed better results (*P*<.05), (see table 1).

**Table 1:** Characteristics of the participants and difference between the groups at the start of the postintervention follow-up study

		Start Follow-up Study				
		Spinal Orthosis n=31		Training n=31		
Variable		Mean ± SD		Mean ± SD		P Value*
**Present height**	(cm)	160.5±7.8		159.9±8.4		.779
**Height young**	(cm)	166.2±5.7		165.6±6.3		.668
**Weight**	(kg)	66.9±14.3		58.6±7.9		.006†
**Back muscle extensor strength mean**	(N)	81.7±41.3		72.8±37.3		.400
**Back muscle extensor strength max**imum	(N)	93.4±46.4		87.9±44.2		.650
**FVC**‡	(L)	2.7±0.7		2.7±0.7		.912
**Gait speed 30 m**	(s)	26.9±11.9		23.6±6.2		.179
**Grip strength right**	(kg)	20.0±5.8		19.8±5.9		.897
**Grip strength left**	(kg)	18.3±5.8		19.1±5.6		.597

Variable		Median	(IQR)	Median	(IQR)	*P* Value§
**One**-**leg standing right, eyes open**	(s)	4	(2-21)	11	(5-30)	.042†
**One**-**leg standing left, eyes open**	(s)	4	(2-13)	18	(5-30)	.004†
**One**-**leg standing right, eyes closed**	(s)	2	(0-4)	2	(1-5)	.091
**One**-**leg standing left, eyes closed**	(s)	1	(0-3)	2	(1.5)	.085
**Tandem standing eyes open**	(s)	26	(2-30)	30	(7-30)	.182
**Tandem standing eyes closed**	(s)	3	(0-8)	5	(2-17)	.048†
**Tandem walking forward**	(steps)	13	(0-15)	15	(4-15)	.305
**Tandem walking backward**	(steps)	15	(0-15)	14	(6-15)	.775
**Visual analog scale back pain, recent**	(mm)	9.5	(0-55)	7	(0-37)	.522
**Visual analog scale** **back pain, last week**	(mm)	39	(19-60)	29	(17-42)	.182
**Borg CR-10 back pain, recent**		1.5	(0-3)	1	(0-2)	.373
**Borg CR-10 back pain, last week**		3	(2-4)	3	(2-3.5)	.642
**EuroQol Health**	(mm)	65	(40-80)	61.5	(50-80)	.457

Abbreviations: FVC, forced vital capacity; IQR, interquartile range.

⁎ *t* test was used for analysis of difference between the groups with normally distributed data.

† *P*<.05.

‡ *P*<.05.

§ Mann-Whitney *U* test was used for analysis of data with skewed distribution.

All participating women reported continued use of the spinal orthosis and that they had continued to train to a varied extent at the follow-up. After 6 months, there were no significant differences between the spinal orthosis group and the training group on any of the variables. Furthermore, no significant difference was shown on any variable within the spinal orthosis group or the training group at the follow-up after 6 months. Back extensor strength had not decreased, experienced back pain had not increased, and balance had not worsened; physical activity and walks were at the same level. Analysis of changes within the spinal orthosis group and within the training group as well as between the groups are shown in table 2.

**Table 2:** Change within the spinal orthosis group and the training group from start to the end of the postintervention follow-up study; difference between the spinal orthosis group and the training group at the end of the follow-up study; analyzed per protocol

Variable	Spinal Orthosis Group		Training Group		Mean Change Within Groups		Difference Between Groups
	Start	6 Months	Start	6 Months	Spinal Orthosis	Training	Spinal Orthosis-Training
Part 1	Mean ± SD	Mean ± SD	Mean ± SD	Mean ± SD	Mean ± SD	Mean ± SD	Mean (95% CI)*
Back muscle extensor strength mean (N)	81.7±41.3	80.8±42.6	72.8±37.3	77.5±32.5	5.4±27.8	1.0±20.8	4.4 (−9.10 to 17.93)
Back muscle extensor strength maximum (N)	93.4±46.4	93.7±47.9	87.9±44.2	90.4±36.7	7.6±31.6	−1.4±27.5	9.0 (−7.29 to 25.29)
FVC (L)	2.7±0.7	2.7±0.7	2.7±0.7	2.5±0.8	0.1±0.4	−0.2±0.6	0.3 (0.03 to 0.60)
Gait speed 30 m (s)	26.9±11.9	24.8±11.4	23.6±6.2	22.4±7.2	−1.1±4.4	−1.0±3.5	−0.1 (−2.26 to 2.00)
Grip strength right (kg)	20.0±5.8	19.6±6.0	19.8±5.9	21.1±5.7	−0.5±2.9	0.7±2.1	−1.2 (−2.57 to 0.18)
Grip strength left (kg)	18.3±5.8	17.9±6.5	19.1±5.6	19.8±5.2	−0.4±3.2	0.0±1.7	−0.4 (−1.83 to 0.94)
Part 2	Median (IQR)	Median (IQR)	Median (IQR)	Median (IQR)	Median Change	Median Change	Difference Between Groups (*P* Value)†
One-leg standing right, eyes open (s)	6.0 (2.0-27.0)	6.5 (2.0-30.0)	21.0 (5.0-30.0)	25.0 (10.0-30.0)	0.5	4	0.0 (.947)
One-leg standing left, eyes open (s)	5.0 (3.0-30.0)	9.0 (3.0-30.0)	21.0 (6.0-30.0)	21.0 (3.0-30.0)	4	0	0.0 (.310)
One-leg standing right, eyes closed (s)	2.0 (0.0-6.0)	2.0 (0.0-4.0)	3.0 (1.0-8.0)	3.0 (2.0-7.0)	0	0	0.0 (.402)
One-leg standing left, eyes closed (s)	2.0 (0.0-4.0)	2.0 (0.0-4.0)	4.0 (1.0-6.0)	3.0 (0.0-10.0)	0	−1	0.0 (.933)
Tandem standing eyes open (s)	30.0 (3.0-30.0)	30.0 (5.0-30.0)	30.0 (14.0-30.0)	30.0 (13.0-30.0)	0	0	0.0 (.534)
Tandem standing eyes closed (s)	4.0 (0.0-18.0)	4.5 (0.0-19.0)	5.0 (2.0-30.0)	8.0 (2.0-30.0)	0.5	3	0.0 (.155)
Tandem walking forward (steps)	14.0 (4.0-15.0)	15.0 (2.0-15.0)	15.0 (6.0-15.0)	15.0 (8.0-15.0)	1	0	0.0 (.857)
Tandem walking backward (steps)	15.0 (0.0-15.0)	15.0 (1.0-15.0)	15.0 (7.0-15.0)	15.0 (6.0-15.0)	0	0	0.0 (.854)
Visual analog scale back pain, recent (mm)	9.5 (0.0-55.0)	2.0 (0.0-33.0)	7.0 (0.0-37.0)	11.0 (0.0-20.0)	−7.5	4	−2.5 (.285)
Visual analog scale back pain, last week (mm)	39.0 (19.0-60.0)	35.0 (25.0-64.0)	29.0 (17.0-42.0)	35.0 (18.0-41.0)	−4	6	−9.0 (.389)
Borg CR-10 back pain, recent	1.5 (0.0-3.0)	1.0 (0.0-2.0)	1.0 (0.0-2.0)	1.0 (0.0-2.0)	−0.5	0	0.0 (.605)
Borg CR-10 back pain, last week	3.0 (2.0-4.0)	3.0 (3.0-3.0)	3.0 (2.0-3.5)	3.0 (2.0-3.0)	0	0	0.0 (.791)
EQ5D Health (mm)	65.0 (40.0-80.0)	51.0 (39.0-72.0)	61.5 (50.0-80.0)	69.0 (50.0-80.0)	−14	7.5	0.0 (.569)

Abbreviations: CI, confidence interval; EQ5D, European Quality of life scale; FVC, forced vital capacity; IQR, interquartile range.

⁎ CI represents the mean difference between spinal orthosis and training with respect to change from start to 6-month follow-up.

† Difference between median change from start to 6-month follow-up for spinal orthosis group compared with training group; *P* value from a Wilcoxon-Mann-Whitney test between spinal orthosis group compared with training group with respect to change from start to 6-month follow-up.

## Discussion

In this study we investigated if training or wearing a spinal orthosis voluntarily for 6 months would be sufficient to maintain previous results from an RCT.^12^ The women had previously experienced decreased symptoms after the intervention period with professional support and regular follow-ups that might have motivate their continued training and wearing the orthosis.^12^ Comprehensive information on how and when to use the spinal orthosis is very important according to a qualitative interview study.^13^ The present study showed that training and using the spinal orthosis independently was sufficient not to lose achieved results after 6 months intervention in the RCT. One explanation may be that the women experienced fewer symptoms from the back and experienced benefits of the training. Women who participated and completed the follow-up study had trained for a total of 1 year, and it has been shown in previous research that individuals with osteoporosis have benefits of performing a comprehensive exercise program regularly over a long period of time.^26^

The exercise program in our RCT focused on strength training of the back extensor muscles and the legs, balance training, and weight-bearing exercises for the skeleton. This is also recommended in several studies, which have shown that physical training can improve balance and prevent falls and fractures,^6^^,^^27^, ^28^, ^29^, ^30^, ^31^, ^32^ improve health-related quality of life,^2^^,^^33^ and decrease back pain.^32^ Exercises that load the skeleton affect bone mineral density positively.^34^^,^^35^

In this follow-up study the women maintained the results achieved during the RCT, which may indicate that they continued to exercise and to wear the activating spinal orthosis, probably because the participants experienced the benefits of feeling stronger and better coping with daily activities increasing their motivation. However, a training study of older women with vertebral fractures showed that the adherence to a home exercise program declined over time.^36^ In another study individuals participated in an education program with the aim of preventing osteoporosis and received both a home exercise program and trained voluntarily. The compliance to the home exercise program was low, and the primary reason for not performing the program was lack of motivation.^37^ One explanation of the maintained results in our study can be that the women previously had been wearing the spinal orthosis for 6 months in the RCT.^12^ The motivation to continue using the spinal orthosis probably increases, when it feels easier to perform daily activities. It has been shown in several studies that strong back extensor muscles may facilitate daily life, which can be in concordance with our results.^1^ Women who wore the spinal orthosis have in a qualitative interview study described that they continued to wear the spinal orthosis and that they felt stronger in the back, back pain decreased, and they also experienced improved posture.^13^ The women in our study received introduction and support to perform the training during the RCT. They perceived that it was important to exercise and continued training according to the exercise program. It has been shown in a qualitative study that professional instructions from a physiotherapist, individually or in groups as well as encouragement, is very important to continue training.^38^ Our results showed that wearing a spinal orthosis as well as performing an exercise program, completely voluntarily for 6 months, did not change obtained results from the previous RCT. Further research is needed to be able to generalize our results to a wider group.

### Study limitations

The groups studied were comparable considering that they had previously been randomized to the RCT. In the RCT, 38 women were randomized to the spinal orthosis group and 38 women to the training group. A total of 62 women completed the RCT, 31 women in each group. All 62 women agreed to participate in the follow-up after 6 months, which is a strength of the present study. One limitation of the study may be that the women might have had the feeling that they were participants in a controlled study because they had been asked if they wanted to come for a follow-up visit after another 6 months. This might have influenced the use of the spinal orthosis and performing an exercise program. Another limitation was that the participating women were a heterogenous group. The women had a wide age range, and they had a big variance in back pain and presence of vertebral fractures. All women were living in the same socioeconomic area in Stockholm. Therefore, we cannot generalize the result to other socioeconomic areas.

## Conclusions

The increase in back muscle strength achieved during the intervention in the RCT was maintained after 6 months of voluntary use of the spinal orthosis and training. Estimation of back pain was not influenced. This indicates that older women with osteoporosis and back pain continued to train and use the spinal orthosis.

## Suppliers

a. Spinal orthosis Spinomed; Medi AB.
b. DigiMax; Mecha-Tronic.
c. Flexicurve ruler; Pedihealth AB.
d. Jamar Hand Dynamometer; Medema.
e. SpiroPerfect; Welch Allyn.
f. Stata, version 14; StataCorp.
